# Chemical Modification of Chitosan for Efficient Vaccine Delivery

**DOI:** 10.3390/molecules23020229

**Published:** 2018-01-25

**Authors:** Lei Xing, Ya-Tong Fan, Tian-Jiao Zhou, Jia-Hui Gong, Lian-Hua Cui, Ki-Hyun Cho, Yun-Jaie Choi, Hu-Lin Jiang, Chong-Su Cho

**Affiliations:** 1 State Key Laboratory of Natural Medicines, Department of Pharmaceutics, China Pharmaceutical University, Nanjing 210009, China; xinglei6xl@163.com (L.X.); fyt2256@163.com (Y.-T.F.); zhoutj1993@163.com (T.-J.Z.); gjh_cpu@yeah.net (J.-H.G.); 2 Jiangsu Key Laboratory of Druggability of Biopharmaceuticals, China Pharmaceutical University, Nanjing 210009, China; 3 Jiangsu Key Laboratory of Drug Screening, China Pharmaceutical University, Nanjing 210009, China; 4 Jiangsu Key Laboratory of Drug Discovery for Metabolic Diseases, China Pharmaceutical University, Nanjing 210009, China; 5 Department of Animal Science, College of Agriculture Science, Yanbian University, Yanji, Jilin 133002, China; cuilianhua@ybu.edu.cn; 6 Department of Agricultural Biotechnology and Research Institute for Agriculture and Life Sciences, Seoul National University, Seoul 08826, Korea; tea0353@naver.com (K.-H.C.); cyjcow@snu.ac.kr (Y.-J.C.)

**Keywords:** chitosan, chitosan derivatives, vaccine, DNA, chemical modification

## Abstract

Chitosan, which exhibits good biocompatibility, safety, microbial degradation and other excellent performances, has found application in all walks of life. In the field of medicine, usage of chitosan for the delivery of vaccine is favored by a wide range of researchers. However, due to its own natural limitations, its application has been constrained to the beginning of study. In order to improve the applicability for vaccine delivery, researchers have carried out various chemical modifications of chitosan. This review summarizes a variety of modification methods and applications of chitosan and its derivatives in the field of vaccine delivery.

## 1. Introduction

A vaccine is a biological agent that provides active acquired immunity to a particular disease. A successful vaccine is one of the most cost-effective alternatives for controlling the incidence and mortality of diseases such as influenza [1], cholera [2], bubonic plague [3], hepatitis A [4], yellow fever [5], measles [6], mumps [7], rubella [8], tetanus [9], diphtheria [10], animal disease [11], etc.

Generally, existing vaccines can be divided into two broad groups: living and non-living vaccines [12]. Vaccines can be further divided into four categories: (i) vaccines containing microorganisms that are artificially inactivated by chemicals or heating; (ii) vaccines containing microorganisms that have undergone attenuated culture or directly use relevant immunological substances; (iii) vaccines derived from inactivated toxic compounds, rather than microorganisms, which are referred to as toxoid-based vaccines; and (iv) subunit vaccines, which use a purified fragment of the pathogens that is able to create an immune response [13]. Moreover, a quantity of innovative bacterial vaccines is also in development and in use [13,14]. Over the past two decades, promising DNA vaccine technology, offering simplicity of preparation, and rapid and relatively inexpensive mass production, has become an alternative to traditional vaccines, and has continued to evolve. DNA vaccines can be used to treat human and animal infections, cancer, allergies or autoimmune diseases [15]. The antigenic structure of DNA vaccines produced in vivo is similar to that produced by natural infection, and can induce highly specific humoral and cellular responses [16].

Vaccines, being the primary public health intervention, provide protection against various infectious diseases [17]. Human immunodeficiency (HIV) infection, the progression of which can be slowed down by antiretroviral therapy but cannot be eradicated, causes approximately one million deaths each year. Using successful vaccines can be more effective for controlling infection [18]. Not only infectious diseases, but more and more researchers try to use vaccines rather than chemical materials, radiation and surgery to fight cancers, and have made some breakthroughs. Matejic et al. [19] found through clinical trials that vaccinated glioblastoma patients exhibited significantly prolonged median survival than those who were not immune. In addition, fibrosarcoma is a locally aggressive malignant tumor. Generally, treating fibrosarcoma requires wide excisional surgery, although it is often difficult to remove enough by incision at the tumor infiltration site. Katsuro et al. [20] showed the utility of recombinant Newcastle disease virus as a candidate for tumor therapy, due to its specific immunity.

**Figure 1:**
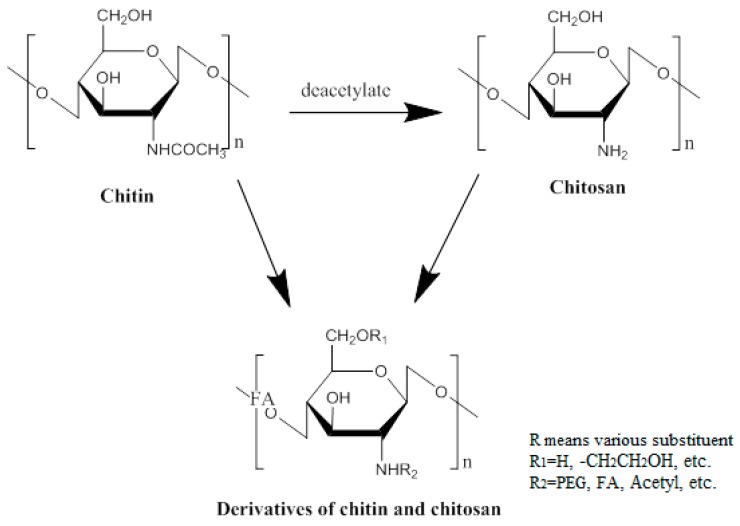
The chemical structures of chitin, chitosan and their derivatives.

For the purpose of effective vaccination, three key factors should be considered: antigen, immunopotentiator and delivery system. Antigens play a role in triggering an adaptive immune response that activates the human immune response. Immunopotentiators act to enhance the immunogenicity and stability of antigens, promoting the synthesis and secretion of antibodies. Delivery systems, as an essential transport medium, make sure that the antigen is targeted to the antigen-presenting cells (APCs) [21]. With the emergence of multiple types of vaccines, the delivery of vaccines also presents a variety of situations. Researchers are committed to designing multifarious targeted delivery systems for the purpose of making the best use of vaccines. For example, nanoparticles (NPs), which can serve as stabilizing vaccine antigens or vaccine adjuvants, such as virus-like particles, liposomes [22], colloidal saponin containing micelles (ISCOMs), polymers and non-degradable nanospheres have been noted as delivery vectors for their ability to activate different elements of the immune system, as well as their good biocompatibility. Some of these NPs can enter APCs through different routes, thereby modulating the immune response to the antigen [23]. Additionally, nature also provides other substances as a gift of vaccine delivery. Chitosan, obtained by deacetylation of chitin, which is widely present in nature (Figure 1), is a slightly pearlescent and translucent flaky white solid. Its basic properties are: (1) chitosan is insoluble in water and alkaline solutions, and soluble in dilute acid, forming a sticky solution. However, in dilute acid, the 1, 4 glycosidic bond of chitosan hydrolyzes slowly, and results in a gradual decrease of the viscosity; (2) chitosan contains hydroxyl, amino and other polar groups, showing good hygroscopicity and moisture retention; (3) chitosan has been used as a drug carrier because it is degraded into a nontoxic substance by lysozyme, releasing the drug slowly, as well as having the property of forming a film; and (4) chitosan can adsorb proteins, amino acids, nucleic acids and other substances by complexation and ion exchange. It is worth nothing that chitosan and its derivatives have good biocompatibility and biodegradability. Not only do the degradation products have no toxic side effects or immunogenicity, and not accumulate, but they also have many unique properties, such as antibacterial, antimicrobial, disease resistance, anticancer, and wound healing. Therefore, it is applied broadly in the pharmaceutical industry compared to conventional excipients, due to these excellent properties. Moreover, the safety of chitosan, as well as its mucosal adsorption-promoting properties, allow it to be developed for vaccine delivery [24]. Over the years, more and more researchers have designed various vaccine delivery systems based on chitosan. They have improved the drawbacks of chitosan by modification to maximize its effects.

In this review, we will summarize the commonly used vaccine delivery systems based on chitosan, and provide an overview of the specific modification of chitosan for vaccine delivery. In detail, we will introduce various improvement programs of chitosan, such as chemical modifications (hydrophilic modification, hydrophobic modification), specific ligand modification (galactose, mannose, peptide), M-cell targeting modification and DNA vaccine. Then, the applications of the modified chitosan will be presented, and we will recommend some research results and applications. Finally, we will offer our own views on these studies and their application prospects.

## 2. Chemical Modification

### 2.1. Hydrophilic Modification

Chitosan has drawn much attention from researchers owing to its nontoxicity and biocompatibility. However, chitosan is only soluble at pH values below 6.5, which dramatically restricts the usage of chitosan in vaccine delivery. To improve the situation of chitosan, water-soluble modifications have been applied; for example, poly (ethylene glycol) (PEG), glycol, poloxamer and alginate have been chosen to conjugate with chitosan. The modifications of the chitosan not only increase the biocompatibility of chitosan but can also enhance water-solubility at the physiological range of 6.8–7.2.

#### 2.1.1. Poly (Ethylene Glycol) (PEG)

**Figure 2:**
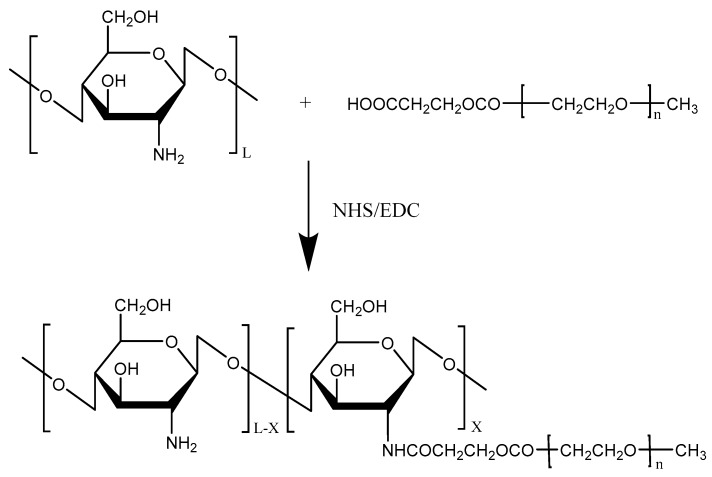
Reaction scheme for the pegylation of chitosan.

Chitosan has been widely used for its excellent biocompatibility and strong mucoadhesive properties for mucosal drug delivery. However, the instability of the chitosan microparticles (CMPs) makes chitosan MPs easily aggregated. Among various water-soluble polymers, PEG was chosen to modify chitosan due to its protein resistance and low toxicity. To overcome the instability of chitosan, Jiang et al. [25] designed and synthesized pegylated chitosan MPs (PCMPs) using PEG as a water-soluble polymer (Figure 2). PCMPs loaded with *Bordetella bronchiseptica dermonecrotoxin* (BBD) with 5.47 μm average particle sizes showed a high BBD loading rate and were more stable than CMPs loaded with BBD, suggesting that BBD-loaded PCMPs could be a promising candidate for BBD vaccine delivery.

#### 2.1.2. Glycol

**Figure 3:**
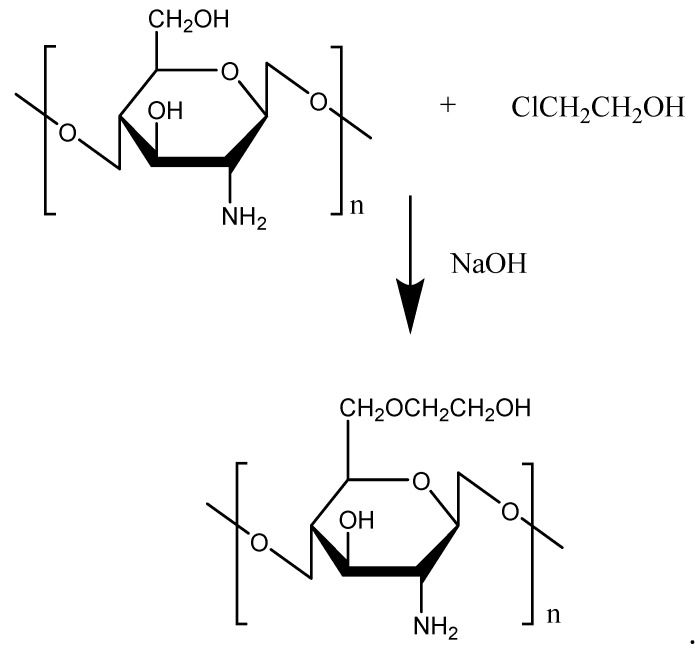
Reaction scheme of glycolated chitosan.

Glycol, due to its strong hydrophilic properties, is often used to modify hydrophobic chemicals to improve water solubility. As a chitosan derivative, glycolated chitosan (GC) possesses low toxicity, high biocompatibility, biodegradability, mucoadhesive properties, and a permeation enhancing effect. The GC was prepared with a reaction of chitosan and chloroethanol (Figure 3). To improve the water solubility of chitosan, bestatin was further conjugated with GC for thymopoietin oligopeptides delivery [26]. The conjugation of bestatin to the GC did not affect the specific interaction between bestatin and aminopeptidase and remarkably protected thymopoietin oligopeptides from enzymatic degradation. Attracted by the excellent performance of GC, Pawar et al. [27] prepared chitosan- and GC-coated PLGA nanoparticles (NPs) (C-PLGA, GC-PLGA). The results verified that the coating of chitosan changed the immune response of PLGA NPs, and GC-PLGA NPs showed better local and systemic uptake, and elicited stronger immune response compared with C-PLGA, which was an indication of the better immune response of C-PLGA NPs by glycol. Additionally, Feng et al. [28] conjugated ethylene diamine tetraacetic acid (EDTA) with GC, and this remarkably inhibited leucine aminopeptidase-mediated degradation of all thymopoietin oligopeptides. Furthermore, Lee et al. [29] used core-shell polymeric NPs composed of a hydrophobic PLGA core and positively-charged GC for transdermal delivery of DNA in epidermal Langerhans cells (LCs). The results showed that the NPs transfected DNA directly into LCs present in the epidermis, and the transfected LCs migrated and expressed the encoded gene products in the skin draining lymph nodes, indicating potential for the usage in immunotherapy and vaccine development. In addition, GC NPs were confirmed to have great potential for the mucosal administration of vaccines [30].

#### 2.1.3. Poloxamer

**Figure 4:**
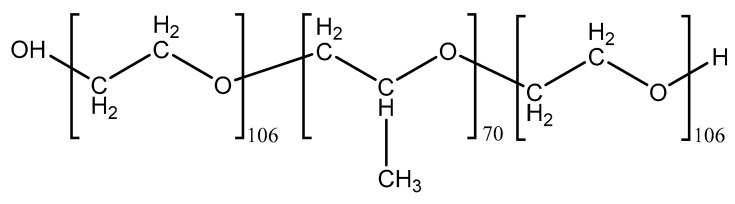
Structure of Pluronic^®^ block copolymer F127.

Poloxamers are copolymers of poly (oxyethylene)-poly (oxypropylene)-poly (oxyethylene) (PEO-PPO-PEO), and possess good drug loading capacity, water-solubility, tolerability, nontoxicity, and controlled release ability due to sol-gel transition [31]. Among them, Pluronic^®^ block copolymer F127 (F127), also known as Poloxamer 408 (Figure 4), a non-ionic hydrophilic triblock copolymer, has been used as a protein stabilizer and immunomodulator to improve the instabilities of vaccine proteins and increase their adjuvanticity [32]. In vitro experiment results demonstrated that *Bordetella bronchiseptica Dermonecrotoxin* (BBD)-loaded CMPs prepared in the presence of F127 showed significantly higher immune-stimulating activities than chitosan MPs, suggesting that the addition of F127 enhanced both the stability of the used vaccine and the uptake of BBD-loaded CMs in immune cells.

### 2.2. Hydrophobic Modification

**Figure 5:**
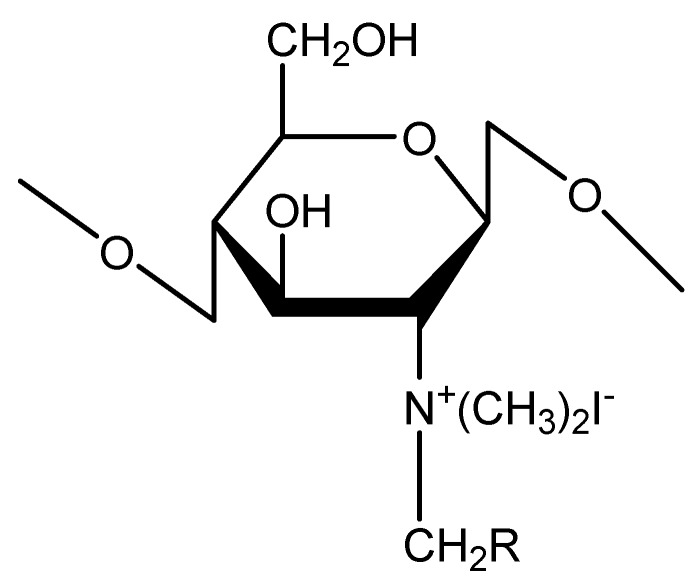
The chemical structure of *N*,*N*,*N*-trimethyl chitosan (TMC).

**Figure 6:**
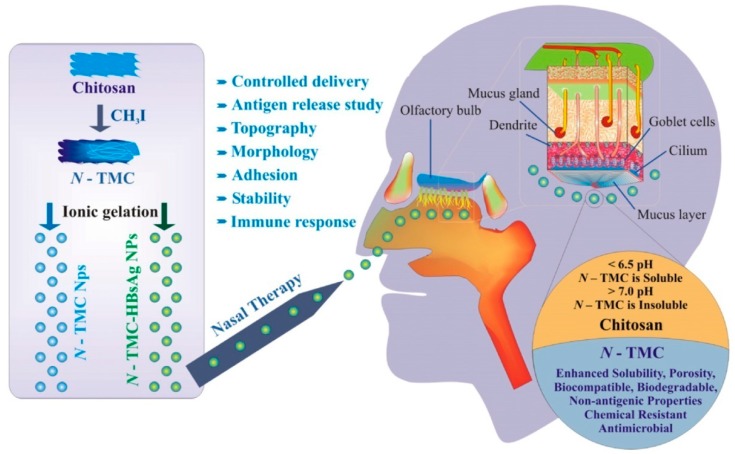
Schematic flow chart representing the preparation of TMC NPs, HBsAg loading and intra-nasal delivery of HBsAg loaded TMC NPs. The nasal delivery and transportation mechanism of vaccine-loaded NPs in the nasal septum area are explained. Anatomy of the nasal area and the advantages of TMC NPs over chitosan are also shown [34].

Chitosan is a very efficient and non-toxic absorption enhancer for both orally and nasally administered peptide-based vaccines [33]. However, chitosan in its protonated form is only water-soluble in acidic environments, due to its pKa value of around 6. Recently, the attention of researchers has been attracted to trimethyl chitosan (Figure 5), because it has excellent water solubility over a wide pH range. Subbiah et al. [34] loaded Hepatitis B virus surface antigen (HBsAg) into *N*,*N*,*N*-trimethyl chitosan nanoparticles (TMC NPs) for a controlled intranasal delivery (Figure 6). The release of HBsAg antigen from HBsAg-loaded TMC NPs reached 93% over a prolonged period (43 days) in vivo. In addition, in vivo immunological study revealed that the adjuvant efficiency of HBsAg-loaded TMC NPs was highly stable for a long time, and was better than the control, suggesting that TMC NPs can be used widely for the treatment of various diseases with a controlled intranasal delivery. Lu et al. [35] investigated the effects of TMC MPs on drug release rate and transport across intestinal epithelial cells. The results showed that the release of drug from drug-loaded TMC MPs did not interact with the intestinal epithelial cells, and did not affect the permeation of the released drug. Maaden et al. [36] coated inactivated polio vaccine (IPV) particles with TMC via electrostatic interactions using pH-sensitive microneedle arrays. The coating of TMC producesd a sufficiently high antigen dose. In addition, the results showed that this method was a useful approach for microneedle-based vaccination.

In addition, methylated *N*-(4-*N*,*N*-dimethylaminobenzyl) chitosan (TMBC), methylated *N*-(4-*N*,*N*-dimethylaminocinnamyl) chitosan (TMCC) and methylated *N*-(4-pyridinylmethyl) chitosan (TMPC) were also prepared to deliver ovalbumin (OVA) for oral vaccination. The results indicated that the TMCC showed the most efficient immune response [37]. The OVA-loaded MPs coated with TMCC by electrospray exhibited the highest in vivo adjuvant activity in both IgG and IgA immunogenicity through oral vaccination. Furthermore, pH-responsive PLGA, chitosan-modified PLGA (CS-PLGA), mannan-modified PLGA (MN-PLGA), and mannan and chitosan co-modified PLGA (MN-CS-PLGA) were used to deliver hepatitis B surface antigen (HBsAg) via nasal administration. The results indicated that MN-CS-PLGA MPs induced stronger humoral and cell-mediated immune responses due to specific interaction of mannose and receptors of the antigen-presenting cells (APCs) compared with others [38].

### 2.3. Specific Ligand Modification

Though poor solubility of chitosan and stability of polyplexes in gene delivery can be overcome by chemical modification of chitosan, the problem of low cell specificity still remains. Thus, with the aim of targeting specific cells and improving cellular uptake efficiency, specific ligands, such as galactose, mannose and various peptides, are commonly conjugated to chitosan-based carriers.

#### 2.3.1. Folate

**Figure 7:**
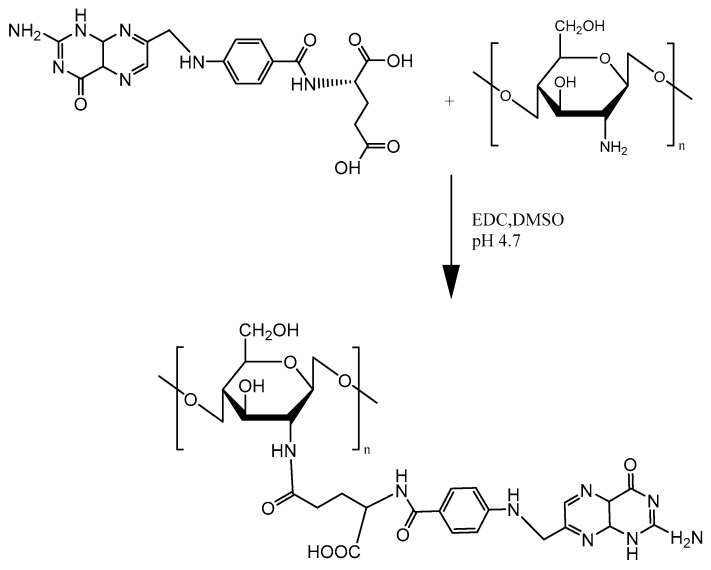
Schematic representation of folic acid-chitosan conjugation.

**Figure 8:**
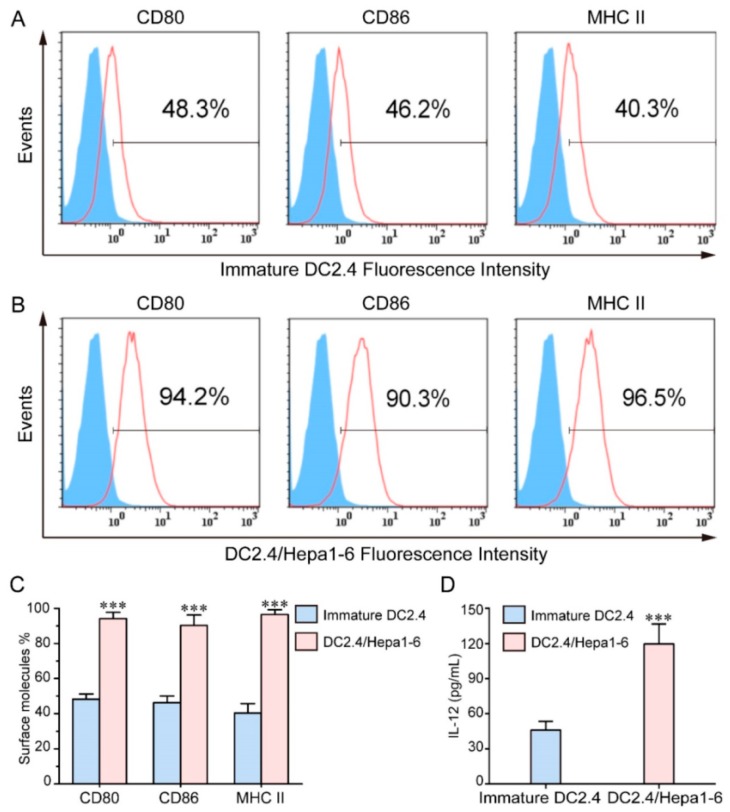
Expression levels of CD80, CD86 and MHCII. (**A**,**B**) Flow cytometry analysis; (**C**) Quantitative analysis; (**D**) The levels of IL-12 (*** *p* < 0.001) [40].

Folic acid (FA) is appealing as a ligand for targeting cell membrane and allowing nanoparticle endocytosis via the folate receptor (FR) for higher transfection yields. Mansouri et al. [39] was the first to describe the technical details of FA-CS preparation (Figure 7). Since then, FA has been commonly used to modify chitosan due to the overexpression of folate receptors on cancer cell surfaces. Hu et al. [40] utilized FA-CS NPs to make polymer complexes with mouse interferon-induced protein-10 (mIP-10) gene and combined them with dendritic cells (DC)/tumor cell fusion vaccine to battle against hepatocellular carcinoma (HCC). As shown in Figure 8, the combination therapy significantly increased tumor-specific IFN-γ responses while reducing myeloid-derived suppressor cells (MDSC) in mouse local tumor, spleen and bone marrow. Furthermore, the combination therapy effectively inhibited the proliferation and growth of tumor cells, while prolonging the overall survival of mice.

#### 2.3.2. Mannose

**Figure 9:**
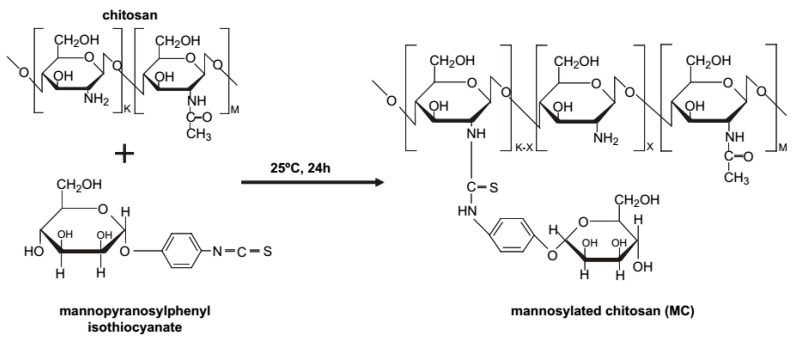
Proposed reaction scheme of MC [41].

**Figure 10:**
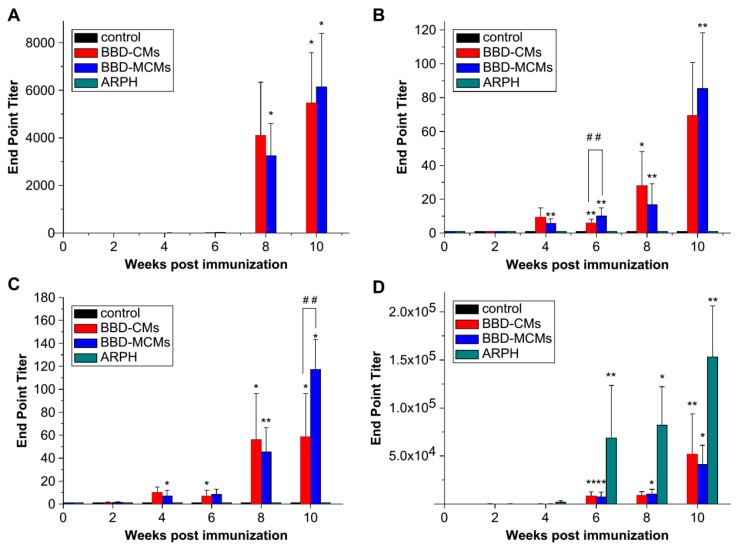
Anti-BBD IgA levels in (**A**) nasal wash; (**B**) saliva (data are means ± standard deviations, *n* = 3). A significant difference between untreated and immunized groups was expressed as * *p* < 0.001, significant differences between untreated and immunized groups were expressed as * *p* < 0.001 and ** *p* < 0.05 and between BBD–CMs and BBD–MCMs groups as ## *p* < 0.05; (**C**) anti-BBD IgA levels in serum; (**D**) anti-BBD IgG levels in serum (data are means ± standard deviations, *n* = 3). Significant differences between untreated and immunized groups were expressed as * *p* < 0.001 and ** *p* < 0.05 and between BBD–CMs and BBD–MCMs groups as ## *p* < 0.05 [41].

Mannose receptors are highly expressed on dendritic cells and macrophages, playing a vital role in immune responses. Thus, polymers with mannosylation can efficiently bind to macrophages via specific interaction with mannose receptors, resulting in enhanced cellular uptake, further promoting antigen presentation and T cell activation. Currently, mannosylation has been widely applied for the modification of drugs and delivery systems to augment immunogenicity. Jiang et al. [41] prepared mannosylated chitosan microspheres (MCMs) (Figure 9) to encapsulate *Bordetella bronchiseptica* antigens containing *dermonecrotoxin* (BBD) for a vaccine delivery platform. The binding ability of BBD-MCMs with murine macrophages (RAW 264.7 cells), on which surface mannose receptors are overexpressed, was confirmed by fluorescence confocal microscopy. In vivo experiments revealed much higher BBD-specific IgA antibody responses induced by BBD-MCMs than that induced by BBD-loaded CMs both in saliva and serum (Figure 10), implying the significant impact of mannose moieties in enhancing immune-stimulating activities via a specific interaction between mannose groups and mannose receptors. Additionally, glucomannosylated chitosan nanoparticles (GMC-NPs) were developed to deliver tetanus toxoid (TT) using the tandem cross-linking method developed by Harde et al. [42]. The obtained GMC-NPs acted both as a vaccine carrier and immunopotentiator for TT. With the help of clathrin- and receptor-mediated endocytosis, higher intracellular uptake was realized by GMC-NPs in Raw 264.7 cells and Caco-2 cells. Compared with the controls, GMC-NPs elicited significantly higher mucosal, humoral and cellular immune response, which is difficult to achieve simultaneously with commercial TT vaccine. In addition, Yao et al. [43] utilized MC to condense anti-GRP DNA vaccine (pGRP). A subcutaneous prostate carcinoma model was established to evaluate in vivo efficacy. The results showed that the level of anti-GRP IgG in mice treated with MC/pGRP was significantly higher than that treated with C/pGRP NPs (*p* < 0.01). Moreover, the growth of tumor cells was suppressed via immunization with MC/pGRP NPs.

### 2.4. M-Cell Targeting Modification

#### 2.4.1. Characteristics of M Cells

The M cells dispersed among epithelial cells can be found in various mucosa-associated lymphoid tissues (MALT), such as nasal-associated lymphoid tissue (NALT), gut-associated lymphoid tissue (GALT) and bronchus-associated lymphoid tissue (BALT) [44], although the M cells in the GALT are very important, because they play a key role in regulating gastrointestinal (GI) tract infection and immunity [45].

The M-cells in the GALT are located on the follicle-associated epithelium (FAE), which lies above the Peyer’s patches in the ileum and jejunum of the small intestine [46]. Also, they uptake and deliver antigens across mucosal epithelia to the underlying lymphoid tissues to generate protective immune responses through transcytosis [47]. Interestingly, the proportion of M cells in the FAE is species-dependent [48]. The percentage of M cells out of the total cells of FAE for the rabbit, rodent and human is around 20%, 10% and 5%, respectively. It has been reported that they originate from stem cells in crypts located between a villus and a Peyer’s patch dome [49], although the origin of M cells is still controversial. 

#### 2.4.2. Targeting Ligand

##### Peptide Ligand

**Figure 11:**
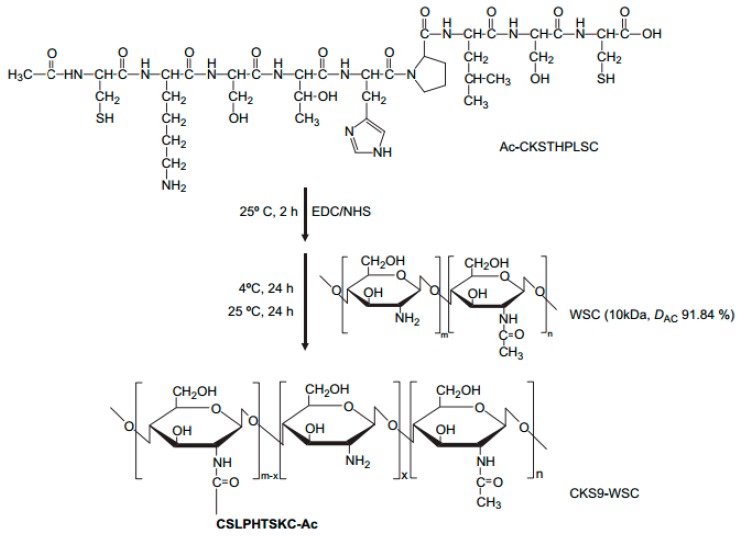
Proposed reaction scheme for synthesis of CKS9-conjugated water-soluble chitosan (CKS9-WSC). CKS9 peptide was chemically conjugated to water-soluble chitosan through the amide linkage between amino group of WSC and carboxylic group in the C-terminal of the CKS9 peptide using NHS/EDC coupling agents [52].

**Figure 12:**
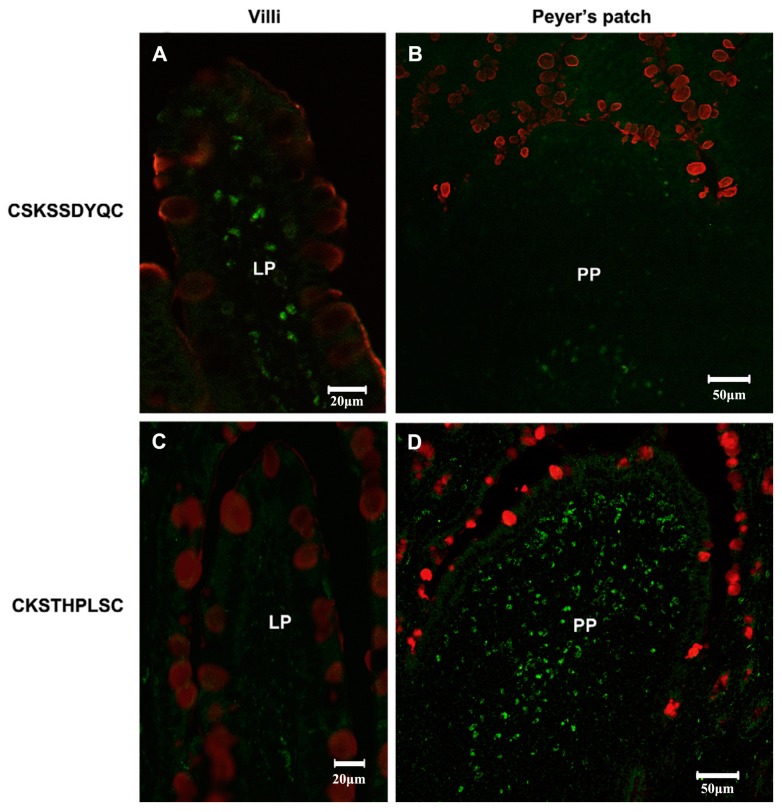
In vivo localization of CKS9 compared with CSK9 in rat small intestinal tissues. Chemically synthesized CKS9 or CSK9 was injected into closed ileal loops, and their tissue-specific localization was monitored using fluorescence microscopy; (**A,B**) In vivo localization of CKS9 peptides in PP and Non-PP; (**C,D**) In vivo localization of CKS9 peptides in PP and Non-PP. Green and red fluorescent signals in each panel indicate the location of the peptides and mucus layer in rat small intestinal tissues (closed ileal loops), respectively. Scale bars indicate 50 µm in PP and 20 µM in Non-PP [52].

**Figure 13:**
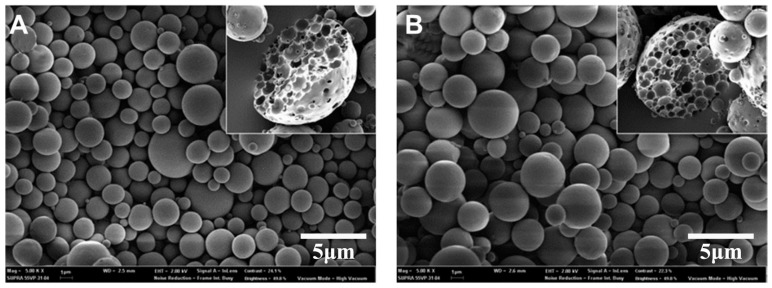

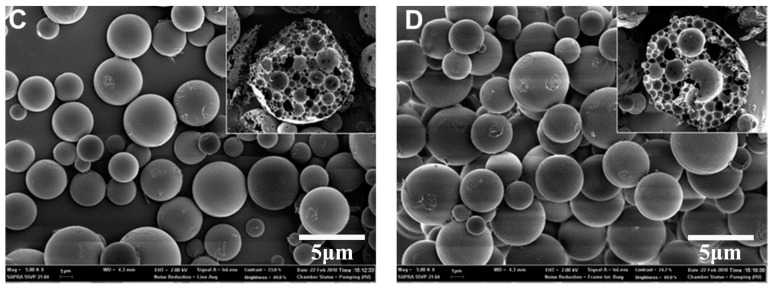
FE-SEM image of the surface and internal morphologies of the PLGA MPs (**A**); WSC-PLGA MPs (**B**), BmpB-PLGA MPs (**C**) and BmpB-WSC-PLGA MPs (**D**) [53].

**Figure 14:**
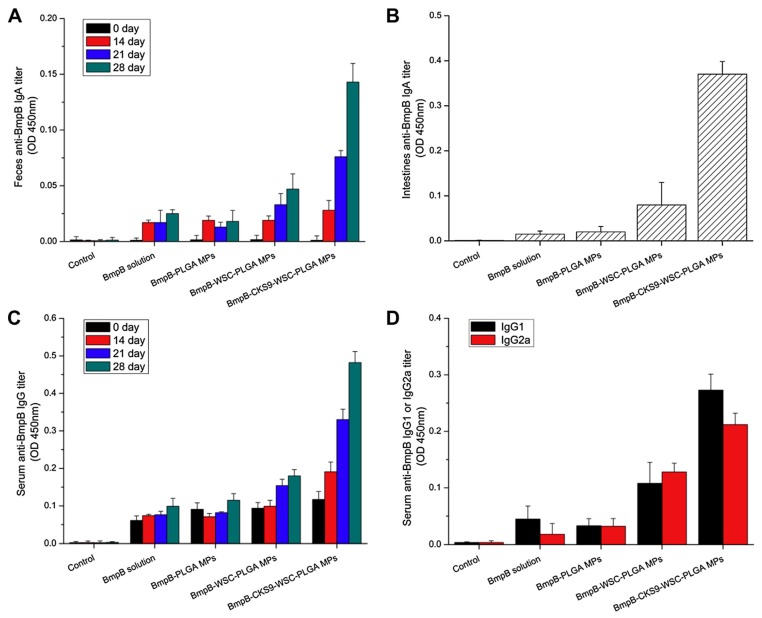
BmpB-specific immune response after oral administration. Anti-BmpB IgA levels in feces (**A**) and intestine (**B**), anti-BmpB IgG levels in serum (**C**) and anti-BmpB IgG subclass antibody (IgG1 and IgG2a) (**D**) levels were measured using ELISA [53].

**Figure 15:**
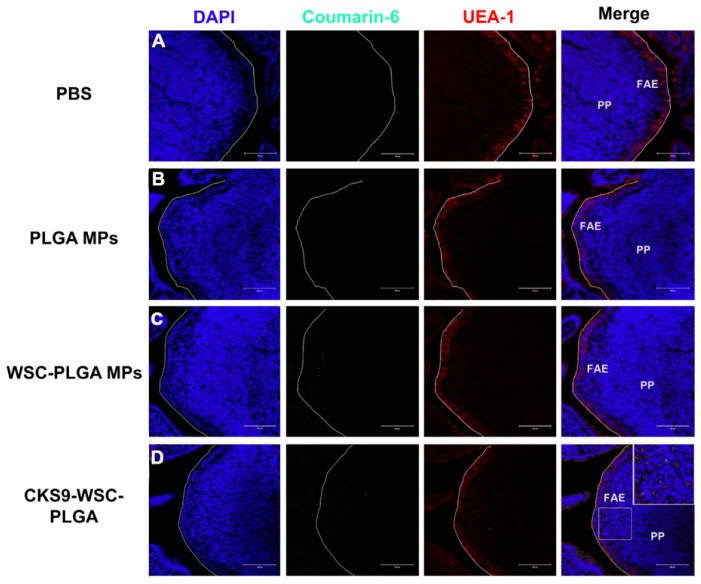
In vivo localization of fluorescent CKS9-WSC-PLGA MPs on the mice Peyer’s patch region 1 h after an injection into closed ileal loops; (**A**) PBS, (**B**) PLGA MPs, (**C**) WSC-PLGA MPs and (**D**) CKS9-WSC-PLGA MPs (scale bar = 100 µm) [53].

The peptides have been seen as attractive as specific ligands because they can be easily synthesized by chemical methods on a large scale [50], and peptide ligands and their cell receptors accumulate in coated pits through a receptor-ligand specific interaction [51]. Generally, there are two kinds of method for screening peptide ligands: chemical synthesis and phage display. Yoo et al. [52] screened M cell-targeting nine-peptide ligands of CKSTHPLSC (CKS9) by the phage display technique, and confirmed the target specificity by an in vitro transcytosis assay and in vivo assay. The CKS9 was conjugated to water-soluble chitosan (Figure 11), and CKS9-conjugated chitosan nanoparticles (CKS9-CNs) prepared by ionic gelation were accumulated into Peyer’s patches regions in greater amounts than chitosan nanoparticles (Figure 12), which suggests that the CKS9-CNs are effective vaccine carriers. They also conjugated CKS9 with chitosan to coat porous PLGA microparticles to deliver *Brachyspira hyodysenteriae* (BmpB) as a swine dysentery vaccine, and the porous surface and internal morphologies of BmpB-PLGA and BmpB-WSC-PLGA MPs were observed to be spherical shapes (Figure 13) [53]. It was also found that oral immunization of BmpB-loaded PLGA microparticles coated with CKS9-conjugated chitosan in mice showed elevated secretory IgA responses in the mucosal tissue and systemic IgG antibody responses (Figure 14) owing to the M-cell targeting and transcytosis ability of CKS9-conjugated chitosan-coated PLGA microparticles (Figure 15).

Similarly, Ye et al. [54] conjugated C terminal 30 amino acids of clostridium perfringens enterotoxin (CPE30 peptide) as the M-cell targeting ligand with chitosan to deliver the coxsackievirus B3 (CVB3) predominant VP1 DNA vaccine in mice. Oral immunization of CPE 30-conjugated chitosan/VP1 DNA vaccine nanoparticles significantly increased specific fecal SIgA level and augmented mucosal T-cell immune responses against CVB3-induced myocarditis due to the M-cell targeting ability of CPE 30.

##### Lectin-Mediated Targeting

**Figure 16:**
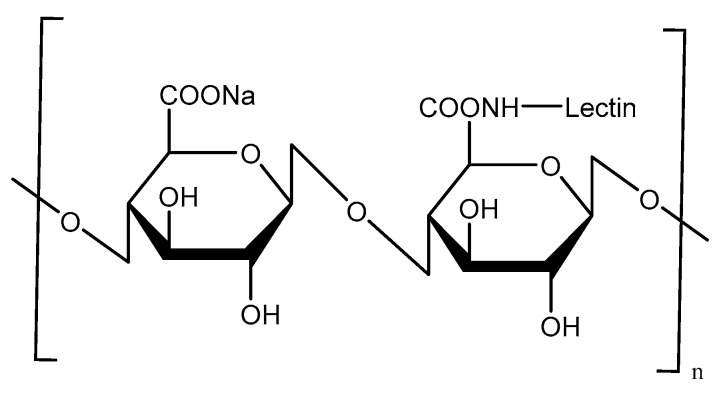
Scheme of UEA-1-conjugated alginate-coated chitosan.

Lectins, as naturally originating proteins, have high affinity with the carbohydrate residues present on cell surface proteins or lipids [55]. The most-studied lectin is ulex europaeus agglutinin1 (UEA-1), as the α-l-fucose-specific lectin. The UEA-1 has been used as a specific ligand for targeted delivery to the M-cells because it binds to α-l-fucose residues expressed on the apical surface of mouse M-cells [56]. Malik et al. [57] prepared UEA-1-conjugated alginate-coated chitosan NPs by ionic gelation to deliver bovine serum albumin (BSA) as a model vaccine in Balb/c mice (Figure 16). Oral immunization of BSA-loaded UEA-1-conjugated alginate-coated chitosan NPs induced mucosal immune responses compared to aluminum hydroxide gel-based conventional vaccine, although M-cell targeting using UEA-1 is mainly restricted to mouse models, because the targets are not present in human M-cells.

Similarly, Kallr et al. [58] conjugated wheat germ agglutinin (WGA), another lectin, with chitosan microspheres (CMs) to load a reduced brominated derivative of noscapine (Red-Br-Nos) to increase the anti-inflammatory response in dextran sodium sulfate (DSS)-induced colitis model mice. The results indicated that the surface bioadhesive properties of WGA-conjugated CMs loaded with Red-Br-Nos promoted affinity toward colon mucin cells in simulated colonic fluid (SCF: pH 7.2) more than CMs loaded with Red-Br-Nos, and WGA-conjugated CMs loaded with Red-Br-Nos remarkably attenuated the DSS encouraged neutrophil infiltration and pro-inflammatory cytokine production in mice compared with CMs loaded with Red-Br-Nos, although it is not clear whether the increased cytokine production in CMs uptake induced by this WGA was attributable to M-cells, because WGA is not specific for mouse M-cells [59].

## 3. DNA Vaccine

**Figure 17:**
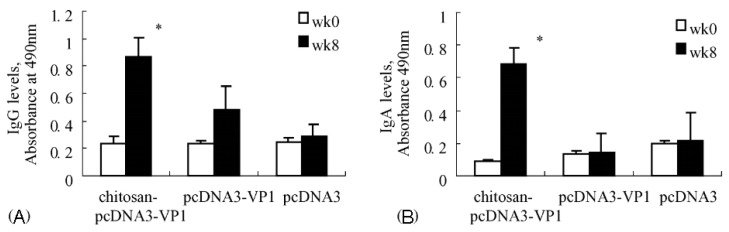
CVB3-specific antibody responses raised by chitosan-pcDNA3-VP1 vaccine. (**A**) Anti-CVB3 IgG responses in serum samples; (**B**) Anti-CVB3 IgA responses in fecal extracts (* *p* < 0.05) [60].

Induction of an immune response by administrating DNA vaccine provides an alternative to other types of vaccination. DNA vaccine-encoded proteins mimic the antigens expressed after viral infection, hence possessing the advantages of safety over live attenuated vaccines and further improving the activation of the immune system. Xu et al. [60] prepared chitosan-DNA vaccine complexes to induce CVB3 specific immune responses via intranasal administration. As shown in Figure 17, after immunization with chitosan-DNA (pcDNA3-VP1) encoding VP1 on mice, much higher levels of mucosal secretory IgA and serum IgG were produced when compared with groups immunized with pcDNA3 or pcDNA3-VP1. Additionally, 42.9 percent of mice treated with chitosan-pcDNA3-VP1 were protected against lethal CVB3 challenge, and the viral load that happened after acute CVB3 infection was also significantly reduced. Zhou et al. [61] developed a novel approach for delivering HBV DNA vaccine more efficiently into APCs via PEI/DNA complexes entrapped in MC microspheres. Compared with naked DNA, enhanced antibody responses were realized by MC microspheres at all doses and time points evaluated. In addition, the time required for detectable antibody responses following immunization was shortened by two weeks with MC microspheres. Similarly, for the purpose of providing pDNA vaccines with an adjuvant-equipped carrier system, Heuking et al. [62] modified chitosan with a Toll-like receptor (TLR-7) agonistic moiety via a PEG linker. The results showed that TLR-7 agonist-modified chitosan significantly increased the interleukin-8-related immune stimulatory ability in human THP-1 macrophages compared with controls.

**Figure 18:**
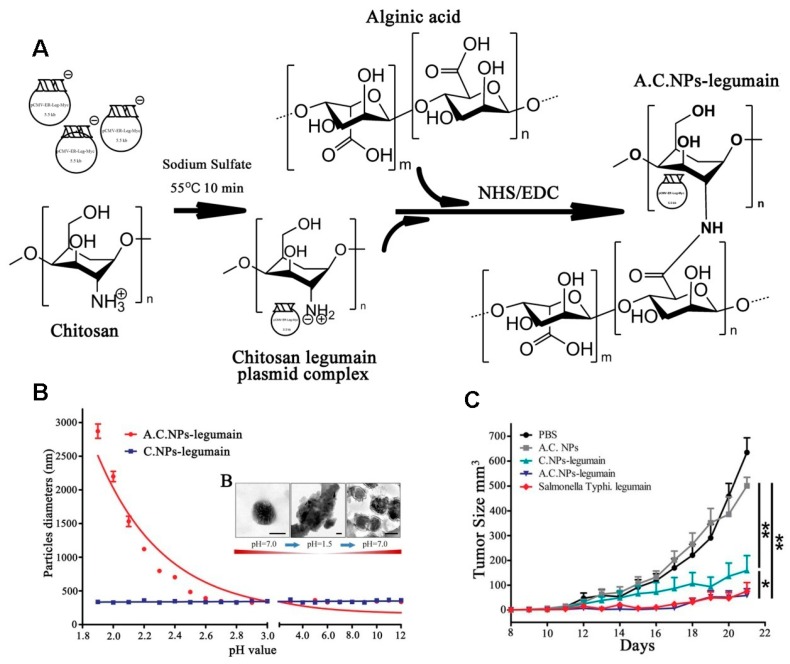
(**A**) Illustration of AC NPs-legumain synthesis; (**B**) Nanoparticles were treated in different acidity levels (pH 1.8, 12) for 2 h: A C NPs-legumain and A C NPs-legumain particle diameter and zeta potential measurements at 37 °C; (**C**) Tumor growth curves. Data are presented as mean ± SD (* *p* < 0.05, ** *p* < 0.01) [65].

Chitosan NPs have been used as a DNA vaccine carrier due to their excellent capacity to enhance mucosa absorption and stability. However, the DNA degradation in the gut has hampered the development of oral DNA vaccinations. Alginate is a natural linear unbranched polysaccharide composed of 1, 4-linked copolymer of β-d-mannuronic acid and α-l-guluronic acid residues [63], and it can be used as oral delivery of DNA vaccine due to its stability in pH 1.2 (simulated gastric fluid) [64]. Liu et al. [65] designed an oral delivery for legumain DNA vaccine using alginate-coated chitosan nanoparticles (AC NPs), as shown in Figure 18A. The results showed that AC NPs were aggregated, having micrometer sizes after forming complexes with DNA vaccine in pH < 2.7, while dispersing into nanoparticles with an increase in pH (Figure 18B), which was an indication of the degree of pH dependence of the complexes. AC NPs have also been used to inhibit the tumor in an orthotopic breast cancer model, and it was found that the complexes showed excellent tumor inhibition (Figure 18C). These studies suggest that alginic acid-coated chitosan NPs can be efficient and safe carriers for the oral delivery of DNA vaccines.

## 4. Conclusions and Future Perspectives

Chitosan and its derivatives hold enormous promise both as adjuvants and delivery vehicles, especially in the forms of micro- and nanoparticulates. This review has explored and evaluated various modifications of chitosan that could help to deliver vaccines more efficiently into APCs, which in turn could induce higher immune responses. Furthermore, after functional modification, multiple properties of chitosan have been substantially improved, such as increased stability, membrane permeability, mucoadhesivity and controlled release behavior, revealing that they are promising vaccine carrier systems. Specific ligands, like mannose, have been conjugated with chitosan derivatives for specific interactions with desired cell types to achieve targeted vaccine delivery. Despite these advantages, many challenges, such as irregular distribution and low physical stability, still hinder the commercialization of chitosan and should be carefully solved in future application. Nevertheless, chitosan and its derivatives have enough potential to encourage researchers to continue investigations in the field of vaccine delivery.
